# panomiX: Investigating mechanisms of trait emergence through multi-omics data integration

**DOI:** 10.1016/j.plaphe.2025.100131

**Published:** 2025-10-11

**Authors:** Ankur Sahu, Dennis Psaroudakis, Hardy Rolletschek, Kerstin Neumann, Ljudmilla Borisjuk, Axel Himmelbach, Kalyan Pinninti, Dominic Knoch, Nadine Töpfer, Jędrzej Szymański

**Affiliations:** a Leibniz Institute of Plant Genetics and Crop Plant Research (IPK), 06466 Seeland, Germany; b Forschungszentrum Jülich, Institute of Bio- and Geosciences (IBG-4 Bioinformatics), CEPLAS, BioSC, Jülich, Germany; c Institute for Plant Sciences, Cluster of Excellence on Plant Sciences (CEPLAS), University of Cologne, Zülpicher Str. 47b, 50674, Cologne, Germany

**Keywords:** Multi-omics integration, Machine learning, Heat stress, High-throughput phenotyping, Explainable toolbox

## Abstract

Complex omics approaches and high-throughput phenotyping generate large, heterogeneous datasets that make linking molecular signatures to plant traits challenging. To address this challenge, here we introduce panomiX, a user-friendly toolbox for multi-omics integration, designed to enable non-experts to apply advanced computational methods with ease. PanomiX automates data preprocessing, variance analysis, multi-omics prediction, and interaction modeling through machine learning, revealing meaningful molecular interactions and synergies. We applied panomiX to a tomato heat-stress experiment combining image-based phenotyping, transcriptomics, and Fourier-transform infrared spectroscopy data, with the aim of identification of condition-specific, cross-domain relationships between gene expression, metabolite levels, and phenotypic traits. Our approach identified a network of such connections, with those linking photosynthesis traits with stress-responsive kinases in elevated temperatures among most significant ones. By simplifying complex analyses and improving interpretability, panomiX offers a platform to accelerate the discovery of trait emergence in plants and select specific candidate genes based on multi-omics analyses.

## 1 Introduction

Recent advances in high-throughput plant phenomics have greatly expanded both the scale and depth of acquired data [1,2]. Phenomics data are often combined with molecular profiling, including transcriptomics and metabolomics [[3], [4], [5]], for instance in GWAS experiments [6], time series [7], or genotype-contrast studies [8]. These combined approaches provide valuable insights into the genetic and molecular basis of trait emergence [9,10]. A prominent example is plant stress resilience, which is shaped at multiple levels: from genetic variation, through gene regulation, to metabolism and biophysical processes [11]. Several studies have shown that profiling these molecular layers (i.e., endophenotypes) can help explain the mechanisms of plant stress responses (summarized in Table 1). Nevertheless, integrating and interpreting such diverse datasets remains a major challenge.

**Table 1:** Examples of recent multi-omic studies related to plant stress phenotypes.

Plant	Omics	Stress	Trait predicted	References
*Zea mays* L.	Transcriptomics	*Ustilago maydis*	Control and Biotic stress	Nazari et al. (2023) [12]
*Solanum lycopersicum plants (cv Micro-Tom)*	Phenomics, Ionomics, Transcriptomics, and Metabolomics	Salinity, Heat, and Salinity + Heat	Stress treatment group	Pardo-Hernández et al. (2024) [13]
*Solanum tuberosum*	Transcriptomic, Metabolomics, and Proteomics	Heat, Drought, and Waterlogging	Control and Abiotic stress	Zagorščak et al. (2025) [14]
*Arabidopsis thaliana*	Transcriptomics	*Botrytis cinerea, Sclerotinia sclerotiorum, and Pseudomonas syringae*	Disease severity	Sia et al. (2025) [15]
*Zea mays* ssp. *mays*	SNPs, Transcriptomics, and Metabolomics	Salt	Salt-tolerance-related traits	Liu et al. (2025) [16]
*Triticum aestivum*	Metaboolomics	Drought	High-throughput phenotyping	Wonneberger et al. (2025) [17]

**Fig. 1:**
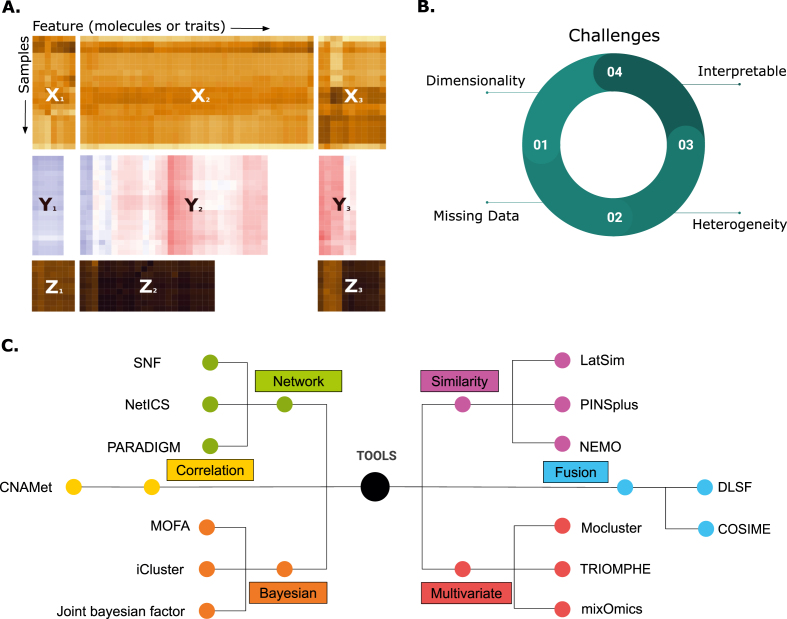
Multi-omic integration - challenges and methods. (A) Omic data sets usually differ in sample size, number of features and variance even if collected in the frame of the same experiment. (B) Different challenges in multi-omics data integration (C) List of available multi-omics data integrative tools.

Multi-omics deals with high-dimensional data [18] (Fig. 1A) that vary in coverage, variance scales, and exhibit batch effects [19] (Fig. 1B). Although many specialized tools exist for analyzing specific omics data types (spanning network-based [[20], [21], [22]], correlation-based [23], similarity-based [[24], [25], [26]], Bayesian [[27], [28], [29]], fusion-based [30], and multivariate methods [[31], [32], [33]]; Fig. 1C), these often fail to integrate different omics layers due to usability constraints, complex workflows, or limited interfaces. Consequently, there is an urgent need for a flexible, comprehensive, and user-friendly solution that can seamlessly integrate diverse omics data types using advanced machine learning techniques.

To address this need, we introduce panomiX, a user-friendly, open-source platform designed for implementing data integration using eXtreme Gradient Boosting (XGBoost) for multi-omic data of varying scales and quality [34]. PanomiX employs a harmonization and scaling pipeline prior to data integration, and accommodates both quantitative and categorical data utilizing cross-predictions between omics layers. XGBoost's inherent ability to handle missing values eliminates the requirement for explicit data imputation. PanomiX provides model interpretability using “SHapley Additive exPlanations” (SHAP) [35], facilitating transparent feature selection, ranking, and visualization. A key feature of panomiX is its capacity to apply user-defined constraints. This enables researchers to test hypotheses on specific biological interactions of interest. This capability extends its utility beyond data integration, allowing for detailed exploration of potential molecular mechanisms between pre-selected sets of phenotype, genotype, and molecular features.

Importantly, panomiX extends beyond classical statistical inference methods, such as ANOVA or correlation analyses, by leveraging XGBoost's strengths in prediction, feature interaction, and ranking. This enables the identification of robust, high-value features across omics layers by capturing nonlinear relationships, which serve as starting points for conventional approaches to address mechanistic questions, such as identifying stress-responsive pathways. In this way, panomiX bridges predictive machine learning with hypothesis-driven inference.

To demonstrate the power of panomiX, we applied it to study the response of tomato seedlings (*Solanum lycopersicum*) to elevated temperatures - an agriculturally relevant process affecting tomato yield both in the greenhouse and field cultivation. Tomato plants are highly sensitive to heat during early growth and later during pollen development, leading to poor germination, inhibited growth, and reduced fruit set [[36], [37], [38]]. At the molecular level, heat stress induces transient gene reprogramming, triggering heat shock proteins (HSPs) and heat shock factors (HSFs), often regulated by MAP kinases and Cysteine-rich Receptor-like kinases (CRKs) [[39], [40], [41], [42], [43]]. Multi-omic approaches capturing transcriptomic, metabolomic, and protein data were crucial to unraveling some elements of these complex regulatory networks [13].

In this study, we analyzed the heat stress response and subsequent recovery of young tomato seedlings under controlled conditions, combining daily high-throughput phenomic profiling with transcriptomics using RNA-seq analysis and biochemical fingerprinting using Fourier-transform infrared spectroscopy (FTIR) at selected time points. FTIR is used here as a proxy for metabolic profiling, a rapid, non-destructive technique that measures the infrared absorbance spectrum of a sample, which reflects the abundance of major functional groups such as lipids, proteins, and carbohydrates. While it does not identify individual metabolites, FTIR captures system-wide biochemical shifts that, when integrated with other omics layers via panomiX, can be linked to specific biological processes. This provided a rapid, global view of metabolic changes that complemented our transcriptomic and phenomic datasets. We describe both common response patterns across all data types and those unique to specific datasets and highlight key interactions among transcripts, metabolic features, and phenotypes uncovered by panomiX. Together, these demonstrate the functionality and versatility of panomiX for multi-omics data integration and analysis.

## 2 Materials and methods

### 2.1 Data preprocessing and analysis

The panomiX platform integrates several specialized R libraries for data harmonization, variance analysis, multi-omics prediction, interaction, and visualization. We provide default data preprocessing methods for transcriptomics and FTIR data. For transcriptomics, we provide a ‘DESeq2-based’ [44] approach, adjusting for factors like sequencing depth and sample composition. For FTIR spectral data, the ‘baseline’ R library [45] is employed to remove background noise, and the ‘signal’ library [46] for Savitzky-Golay smoothing [47] of spectral features without loss of peak sharpness and spectral integrity. Variance analysis is conducted using the ‘irlba’ R library and prcomp function [48], including dimensionality reduction, clustering, and identification of sources of variance. Multi-omics prediction workflows are facilitated by ‘caret’ R library [49], with the built-in framework for hyperparameter tuning and model evaluation. XGBoost is used for predictive modeling, and SHAP and Boruta-SHAP are employed for model interpretability and feature selection [50].

### 2.2 Visualizing insights with interactive plots

The panomiX user interface (UI) uses ‘shinyjs’ [51] to create dynamic interactions, allowing users to control UI element visibility based on their actions, for a streamlined experience. Interactive plots are created with ‘plotly’ [52], enabling users to zoom, pan, and hover over data points for deeper exploration. The ‘DT’ package [53] enables interactive data tables, facilitating sorting and exploration of large datasets. ‘shinycssloaders’ [54] provides visual feedback on application progress during data loading. Additionally, ‘bslib’ [55] offers advanced plot formatting tools, and ‘bsicons’ [56] incorporates intuitive icons, enhancing UI usability and aesthetics.

### 2.3 Source code availability and community collaboration

The panomiX toolbox is developed fully in R [57] and Shiny [58] and deployed on Shinyapps. io https://www.shinyapps.io/. The source code is managed with a GitHub repository connected to the Shinyapps. io via ‘rsconnect’ [59]: https://szymanskilab.shinyapps.io/panomiX/. The source code for the platform is available on GitHub: https://github.com/NAMlab/panomiX-tool. The repository contains all the necessary R scripts for data processing, visualization, and machine learning prediction.

### 2.4 Experimental data and design

Seeds of genotype *Moneymaker* were planted in well-watered soil trays and kept under a plastic cover in a walk-in phytochamber with a day/night cycle of 16/8h and temperatures of 24 °C and 20 °C respectively. The seeds were kept without light for one day, then illumination of 320 μmol per square meter per second (from Whitelux Plus metal halide lamps, Venture Lighting Europe Ltd., Rickmansworth, Hertfordshire, England) was added during the day phase. After a total of 7 days after sowing, evenly germinated and healthy seedlings were transplanted into individual pots (10 cm diameter, 8 cm height) without plastic covers at 60–70 % relative air humidity. After 9 further days of establishment in the pot, the plants were exposed to heat stress at 37 °C/28 °C (day/night) for 6 days followed by a recovery phase at 24 °C/20 °C. Plants were watered once or twice daily by an automated system replenishing evapotranspirated water by weight to ensure the plants do not experience drought stress. All plants were phenotyped daily using an imaging-based high-throughput system and the youngest fully formed leaf pair was harvested (directly put in liquid nitrogen and then stored at −80 °C) for molecular measurements mid-day (between 13:00 and 14:00, light phase was from 06:00–22:00) from individual plants directly before the temperature switch, each day during the heat stress phase and after 1, 2, and 4 days of recovery. Control plants were grown exactly the same way except that they stayed at 24 °C/20 °C throughout the whole experiment. They were sampled at the same respective time points as the heat-treated plants.

### 2.5 Data acquirement

During daily phenotyping, plants were photographed from the top and three side angles in visible and in fluorescent light (excitation: 400–500 nm, emission: 520–750 nm). The images were then analyzed using the Integrated Analysis Platform (IAP) software [60] to yield 138 phenotypic traits for each measurement, as described in Ref. [61]. Next we reduced the 138 phenotypes into a smaller set of non-correlated variables. Using hierarchical clustering, we grouped related phenotypes together. The number of clusters was estimated using PCA, determining the minimal set of clusters, for all of which the first PC described at least 50 % of their variance (representing the cluster co-linearity). Through this process, we narrowed the phenotypes down to 27 key representatives, which we used for model training.

For the molecular assays, the frozen leaves were ground and aliquots subjected to specific measurements: Total RNA was isolated from 70 mg of the ground material using the RNAeasy Plant Mini Kit (QIAGEN) according to the manufacturer's protocol. The construction of sequencing libraries involved the Illumina stranded mRNA Prep Ligation Kit (standard Illumina protocol; Illumina, San Diego, California, USA) and 1 μg DNAse I digested total RNA. Sequencing of equimolar library pools (average size: 332 bp) was performed on an Illumina NovaSeq 6000 device (IPK-Gatersleben), using the XP-workflow and a S2 flowcell (Illumina, San Diego, California, USA). On average, 41 M reads (single reads, 118 cycles) were generated per sample. Reads were then mapped to the ITAG4.1 tomato reference genome (https://solgenomics.net/ftp/tomato_genome/annotation/ITAG4.1_release/) using our rnaseq-mapper pipeline built around kallisto [62], available at https://github.com/NAMlab/rnaseq-mapper; [63].

FTIR analysis of freeze-dried leaf material was performed as in Ref. [64]. Briefly, approximately 2 mg of freeze-dried material was used per sample. Spectra were produced from ATR-FTIR measurements using the INVENIO-S FTIR spectrometer (Bruker Optics, Ettlingen, Germany) with a Globar light source under continuous purging with dry air. ATR absorbance spectra were recorded in the spectral range of 4000-400 cm^−1^ at a spectral resolution of 4 cm^−1^. Each spectrum consisted of 32 co-added scans. As a background reading, the spectrum of the empty ATR crystal was collected prior to measurement and subtracted automatically from each recorded spectrum using the OPUS software (Bruker Optics).

## 3 Results

### 3.1 Data input

**Fig. 2:**
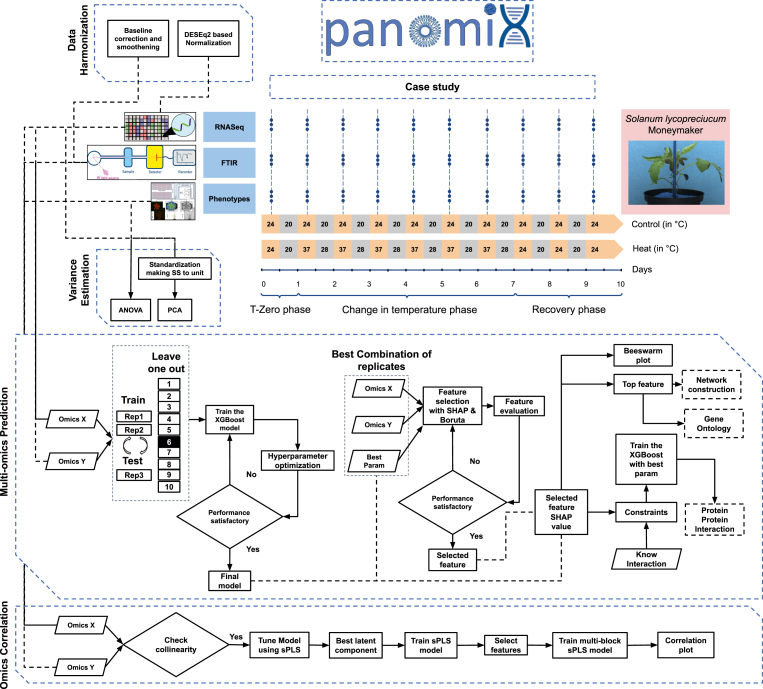
Flow diagram of the panomiX toolbox and the experimental design for the tomato heat stress case study.

Our experiment provided 138 phenotypic variables reduced to 27 uncorrelated phenotype clusters (Fig. S1), 34,688 gene expression values, and 2526 FTIR data points across 10 time points and 2 to 3 biological replicates in two experimental conditions: control growth, and treatment with 6 days of heat stress treatment followed by 3 days recovery phase (Fig. 2).

#### 3.1.1 Tool settings and recommendations

PanomiX works with continuous molecular data, such as normalized RNA-seq counts, protein abundances, metabolite concentrations, or FTIR spectra. Your data should be in a feature matrix format, where: Columns represent biological samples (e.g., individuals or time points). Rows represent molecular features (e.g., genes, proteins, metabolites, or spectral variables). A continuous outcome variable (y) is needed for regression tasks. PanomiX can handle large datasets with tens of thousands of features, but pre-filtering your data is recommended for better performance and computational efficiency. Here's how you can optimize your dataset: filter low-variability features, remove features that show little variation across samples, exclude low-count features for sequencing data (like RNA-seq), and drop features with consistently low counts, remove near-zero variance features which contribute little to the model and can be safely excluded. These steps help reduce the size of high-dimensional datasets, minimize overfitting, and improve the speed of machine learning algorithms like XGBoost during model training and hyperparameter tuning. Before using panomiX, ensure that your datasets are pre-processed and normalized according to the requirements of the specific ‘omics platforms.

While methods such as XGBoost can accommodate *p ≫ n* settings, omics datasets with fewer than ∼50 observations should generally be regarded as exploratory. The effective number of required samples for panomiX depends on factors such as the signal-to-noise ratio, the correlation structure among predictors, and the stability of cross-validation performance. We recommend that readers first examine the variance structure of their data using multivariate approaches (e.g., Principal Component Analysis, PCA) to estimate how many principal components explain the majority of variance. For example, if three PCs capture ∼90 % of the biological variance, a relatively simple model with only a few effective predictors may be sufficient, regardless of the initial dimensionality. When sample size is limited, model robustness could additionally be assessed using learning curves or resampling strategies. Importantly, the number of predictive features retained by the model is constrained by sample size and should remain considerably smaller than *n*.

### 3.2 Data processing and filtering

**Fig. 3:**
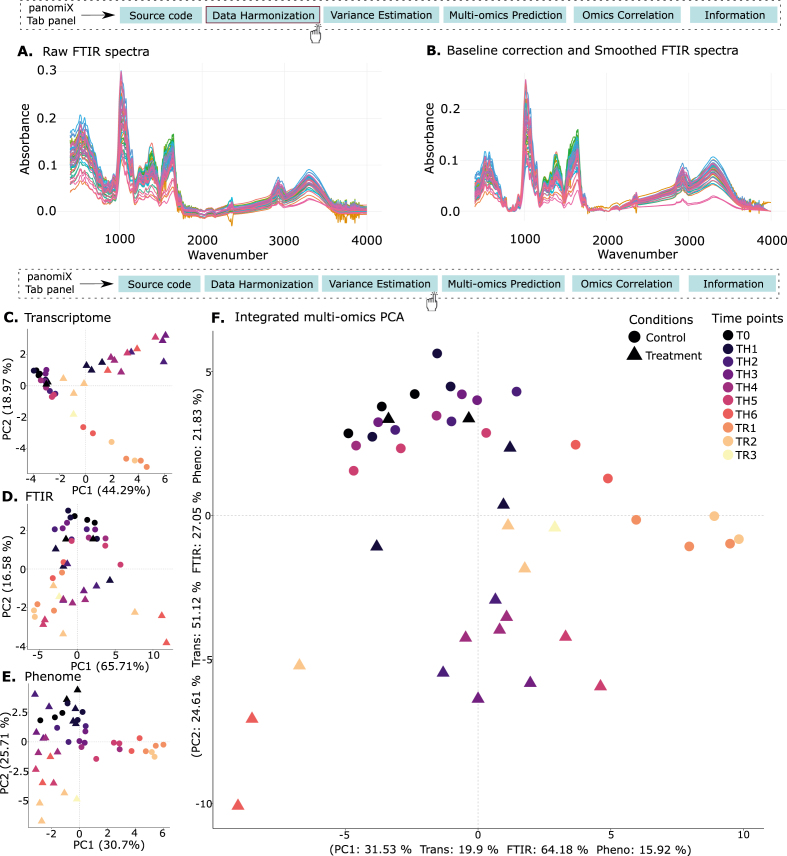
Data harmonization and variance estimation component (A) Raw FTIR spectra (B) Baseline corrected and smoothed FTIR spectra. Individual PCA for each omics data (C) Transcriptome, (D) FTIR, and (E) Phenome. (F) An integrative PCA, where the multi-omic data matrix was combined to identify shared variance components.

The RNA-seq data processing with rnaseq-mapper [https://github.com/NAMlab/rnaseq-mapper] provided a data matrix of estimated TPM values (transcript per million, kallisto estimates; [62]) for 49 samples (both control and treatment), with columns representing samples and rows representing genes-aggregated transcript levels. We used TPM counts without applying the data harmonization component for further analysis. To filter out transcripts with consistently low TPM counts, we retained only those transcripts that have at least one sample with a count of 50 or higher, ensuring that transcripts with TPM counts below 50 across all samples were removed. Ultimately, we used 5479 transcripts for further analysis. Similarly, for FTIR spectral data, we created a data matrix of absorbance values, containing 49 samples (both control and treatment). We selected the absorbance values in the range from 4000 to 400 cm^−1^. We then applied FTIR normalization using the data harmonization component in the panomiX toolbox. For illustration Fig. 3A presents the raw FTIR spectra before preprocessing. After baseline correction and Savitzky-Golay smoothing, the normalized spectra are displayed in Fig. 3B, illustrating the improved signal quality.

#### 3.2.1 Tool settings and recommendations

PanomiX provides standard methods for normalization of transcriptomic data [44]. First, a natural log transformation is applied to (*x*_*ij*_) a raw expression count for gene *i* in sample *j* to stabilize variance and improve comparability (Eq. (1)). Then, the transformed gene expression data is used to determine the average estimation for each gene. Next, non- and sparsely expressed genes are filtered out, allowing us to focus only on stably expressed genes (*G*). The previously log-transformed value of each gene is then subtracted from the respective average, helping identify genes within each sample that have higher or lower expression levels than the average. Subsequently, the average-subtracted values are used to determine the median across genes, which reduces the influence of high-value outliers. Finally, the median values are transformed back from log to normal scale to obtain the final scaling factor (Eq. (1)). The raw read counts are then divided by these scaling factors (Eq. (2)). This log and median-based scaling method effectively corrects batch effects and extreme variations. For consistency, we recommend performing all steps of data normalization from the raw counts within panomiX. However, the platform also accepts normalized transcriptomics data in TPM or CPM counts. For the spectral data, such as those obtained by FTIR, panomiX utilizes the baseline correction on the raw spectrum to remove artifacts and the background noise. Following this, Savitzky-Golay smoothing can be applied to each baseline-corrected spectrum. This step applies a polynomial smoothing filter to reduce high-frequency noise while maintaining essential spectral features.

The scaling factor for sample j, is calculated as:

(1) $$s_{j}=\exp\left({median}_{i\epsilon G}\;\left[\log\left(x_{ij}\right)-\frac{1}{n}\sum_{k=1}^{n}\;\log\left(x_{ik}\right)\right]\right)$$

where *x*_*ij*_ is the raw expression count for gene *i* in sample *j*, *n* is the number of samples, and *G* is the set of genes with finite mean log-expression.

The normalized expression values are then given by:

(2) $$x_{ij}^{norm}=\frac{x_{ij}}{s_{j}}$$

### 3.3 Variance analysis

The exploratory analysis of the multi-omic data variance highlighted both data type-specific, and shared patterns of changes driven by plant development and heat-stress treatment. In the transcriptome PCA (Fig. 3C), PC1 (44 % variance) largely reflects time-dependent changes in gene expression, while PC2 (18 % variance) highlights heat-stress effects. Early-stage controls and T0 heat samples cluster together, indicating minimal transcriptional differences at the start, whereas later-stage heat treatments diverge, revealing progressive stress adaptation. In FTIR absorbance data (Fig. 3D), PC1 (65 % variance) again captures temporal shifts, and PC2 (16 % variance) distinguishes stress from control conditions; notably, mid-stage heat-treated seedlings cluster with recovery-stage controls, indicating partially overlapping metabolic responses. Phenomic data (Fig. 3E) show stronger treatment separation on PC1 (30 % variance) and time progression on PC2 (25 % variance), where early-stage controls resemble T0 heat samples, and later stages separate more distinctly. Integrating all omics layers (Fig. 3F) confirms the interplay of time and treatment: PC1 (31 % variance) is dominated by FTIR and transcriptome signals, clearly separating recovery-stage heat-treated seedlings, while PC2 (24 % variance), driven by transcriptional variation, further distinguishes controls from heat-stressed plants at various stages. These results accentuate the distinct yet complementary biological insights gained by analyzing temporal and stress effects across transcriptomic, metabolic, and phenotypic data.

#### 3.3.1 Tool settings and recommendations

PanomiX supports exploratory data analysis with PCA or ANOVA to identify dominant sources of variance. Before PCA, all datasets are standardized to a unit sum of squares, ensuring comparable total deviations across different data types, but all the relevant dataset-specific normalization steps should be performed beforehand. First, PCA is performed separately on each omics dataset to capture dataset-specific variance patterns. Second, the integrated PCA is conducted on a combined dataset to reveal shared variance and global patterns. In this case each block of omic data is first scaled to a unit sum of squares and then concatenated into a single input. For PCA, the tool automatically handles data centering and scaling. For the ANOVA and plotting the PCA scores, the user needs to upload metadata with at least an ID column (matching the omics data) and respective condition columns if experimental factors are present. PanomiX often exposes differences in resolution and variability among different omic datasets, demonstrating how multi-omics integration can also provide insights into each individual dataset. Notably, these differences should be carefully evaluated, as differences in normalization, coverage and data filtering procedures can confound their biological interpretation.

### 3.4 Multi-omics prediction components

**Fig. 4:**
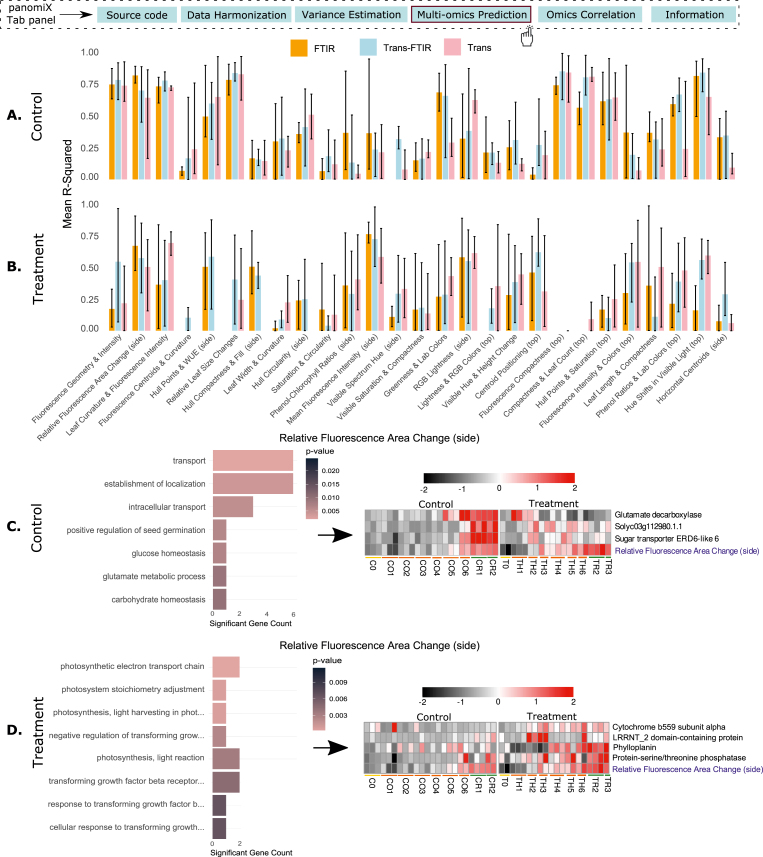
(A) Performance of control samples for transcripts predicting phenotypes, FTIR predicting phenotypes and combined transcripts + FTIR predicting phenotypes (B) Performance of treatment samples for transcripts predicting phenotypes, FTIR predicting phenotypes and combined transcripts and FTIR predicting phenotypes (C) Gene ontology and expression profile for the features selected for Relative Fluorescence Area Change (side) from the control model (D) Gene ontology and expression profile for the features selected for Relative Fluorescence Area Change (side) from the treatment model.

Linking the high-throughput profiling of plant traits with molecular assays such as RNA-seq or metabolomics, can yield valuable biological insights into molecular mechanisms of trait emergence. To explore these connections, we conducted cross-prediction on phenotypes using transcriptomics alone, FTIR alone, and combined transcriptomics + FTIR data, each trained separately on control and treatment samples. This approach enabled identification of condition-specific molecular markers. Model performance, assessed via mean R-squared values across three replicate splits, showed generally higher accuracy in control samples; however, some treatment-based models (e.g., Phenol–Chlorophyll Ratios (side), Mean Fluorescence Intensity (side)) outperformed their control counterparts (Fig. 4A and B). Next, we performed gene ontology (GO) enrichment using ‘topGO’ R package [65] on transcripts with high SHAP values (i.e., highly predictive of phenotype), focusing on models with R^2^ > 0.5 and replicate-model R^2^ > 0.7. This criterion yielded 10 control and 7 treatment models (Fig. S2A and S2B). Certain phenotypes had unique GO enrichments, while others overlapped. For instance, a control model predicting Relative Fluorescence Area Change (side) highlighted Glutamate decarboxylase and Sugar transporter ERD6-like 6 both linked to enhanced photosynthetic capacity [66] (Fig. 4C). Meanwhile, the corresponding treatment model identified cytochrome *b*559 subunit alpha, part of the photosynthetic electron transport chain (Photosystem II), and phylloplanin, which contributes to stress defense via type VI glandular trichomes [67,68] (Fig. 4D). Next, we extended the analysis to FTIR and combined transcriptomics + FTIR models, applying the same performance thresholds (R^2^ > 0.5 overall and R^2^ > 0.7 for individual replicate models). Within this subset, combined transcriptomics + FTIR models improved predictive ability by an average of 10 % (up to 43 %) compared to transcriptomics alone and average of 5 % (up to 24 %) compared to FTIR alone under control conditions. Under treatment conditions, integration yielded an average improvement of 12 % (up to 33 %) over transcriptomics alone and 12 % (up to 40 %) over FTIR alone (Fig. 4A and B). This consistent performance gain demonstrates that multi-omic fusion provides complementary information not captured by single-omic models. Notably, several transcripts and FTIR features were consistently identified as top predictors across all three omics-specific models (Table S2). For example, in the Relative Fluorescence Area Change (side) phenotype (Fig. S3), the transcript-only model pinpointed cytochrome *b*559 subunit alpha and phylloplanin as key contributors (Fig. S3A). In the FTIR-only model, spectra 2002 and 2003 showed strong predictive power (Fig. S3B). The integrated model (transcriptomics + FTIR) retained these same predictors, while also incorporating additional predictors (Fig. S3C), confirming their importance. Further analysis of the transcript-to-FTIR prediction revealed that phylloplanin significantly predicted the 2002 FTIR spectrum (Fig. S3D).

#### 3.4.1 Tool settings and recommendations

Training the XGBoost model requires splitting the data into training and test samples. Users can split datasets either randomly (using a training size slider) or by replicate to ensure consistent grouping of the same replicates, and hyperparameter tuning is automated via the caret method. In the cloud version of panomiX, two main hyperparameters (number of rounds, tree depth) can be configured, with others (learning rate, gamma, subsample, etc.) preset to optimal ranges. The desktop version provides greater flexibility, letting users adjust a wider range of parameters (see the full documentation; https://github.com/NAMlab/panomiX-tool). Model generalization is validated through cross-validation (CV) or leave-one-out cross-validation (LOOCV), and performance is reported using R^2^ and RMSE. A model performance plot (R^2^) is displayed, alongside a feature-importance table that ranks predictors. SHAP values further reveal how individual features positively or negatively influence phenotype predictions, visualized via beeswarm plots for intuitive interpretation. The Boruta-SHAP algorithm is also available for alternative feature selection, with its results shown in a separate beeswarm plot. For biological replicates, we recommend replicate-based splitting (with metadata specifying sample names and replicate identifiers) to improve model consistency and accuracy. Random splitting with an adjustable training size can be used for general evaluation. In the cloud version, tuning the number of rounds and tree depth is crucial for better performance; the desktop version allows deeper parameter control. CV is advisable for robust model generalization, while LOOCV suits smaller datasets. For deeper insights, users can leverage SHAP values and feature-importance rankings to interpret and prioritize key predictors.

### 3.5 Multi-omics prediction with interaction constraints

**Fig. 5:**
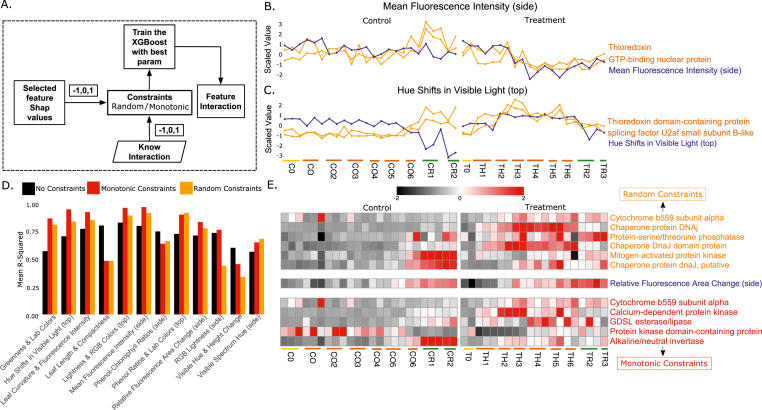
Feature interaction. (A) Illustration of feature interaction as a flow diagram (B) Interaction between the heat stress related (scenario a) transcripts and predictive features from trained model Mean Fluorescence Intensity (side) (C) Interaction between the heat stress related (scenario a) transcripts and predictive features from trained model Hue shifts in Visible Light (top) (D) Performance of treatment samples for transcripts predicting phenotypes (scenario b). Black color bars represent models trained without constraints, red color bars models with monotonic constraints, and orange color bars models with random constraints, respectively. (E) Expression profile for the features selected from the constraints model of Relative Fluorescence Area Change (side) (scenario b). Orange colored features are selected from the random constraints models and red colored features are selected from the monotonic constraints models.

PanomiX can analyze interactions of predictive features identified by the model as well as “known” features provided by users - such as genes of interest - by incorporating both sets into a single prediction framework. It uses SHAP values to show how these features (positively or negatively) influence a given outcome (e.g., a phenotype) and to set constraints for the model (Fig. 5A). In our experiment, these constraints relate to a predetermined list of literature- and annotation-based candidate transcripts. We tested two scenarios: a) evaluation of data-derived candidate transcript set; and b) evaluation of a predetermined list of candidates.

In the first scenario, we tested 57 heat-stress-related transcripts (46 up-regulated, 11 down-regulated; Psaroudakis et al. [63]) alongside the features previously identified by panomiX. Notably, several transcripts showed positive interactions with both the predicted features and the target phenotypes (Table S3). For instance, in a heat-treatment model predicting Mean Fluorescence Intensity (side), GTP-binding nuclear protein (down-regulated under heat stress) maintained its down-regulation relative to controls, aligning with its predictive role. Similarly, in a model predicting Hue Shifts in Visible Light (top), thioredoxin domain–containing protein interacted with splicing factor U2af small subunit B-like, suggesting a joint effect on the phenotype under heat stress (Fig. 5B and C).

In the second scenario, we explored whether stress responses extend beyond heat shock proteins to include kinases and other signaling elements. Transcriptional regulators together with protein kinases constitute key classes of regulatory proteins that govern plant growth, development, and responses to both biotic and abiotic stimuli [69]. In addition, enzymes such as guanylate cyclases, which catalyze the conversion of GTP to cyclic GMP, act as important upstream regulators that activate protein kinase–mediated signaling pathways during abiotic stress responses [[70], [71], [72], [73]]. For that purpose, we compiled 1531 candidate genes (99 guanylate cyclases, 1038 other kinases, 394 heat-related genes) from literature and databases [69,74,75]. We then trained models using no constraints, random constraints, or monotonic constraints (Fig. 5D). Most cases showed improved performance with random constraints over no constraints, and an additional ∼5 % improvement under monotonic constraints. A representative example is a model predicting Relative Fluorescence Area Change (side), where random constraints boosted performance by 5 % compared to no constraints. Key transcripts (e.g., cytochrome *b*559 subunit alpha, protein-serine/threonine phosphatase) interacted with MAP kinase and DnaJ domain proteins (Table S4). Under monotonic constraints, performance rose another 5 %, and cytochrome *b*559 subunit alpha was linked to GDSL esterase/lipase and calcium-dependent protein kinase, both implicated in stress response [[76], [77], [78]].

Overall, these findings highlight panomiX's capacity to reveal significant interactions among user-specified and model-predicted features, offering mechanistic insights into phenotype determination and guiding further experimental validation.

#### 3.5.1 Tool settings and recommendations

By including a user-provided list of relevant (or potentially important) known features, panomiX can determine whether these inputs significantly contribute to the model's outcome or interact with features already identified by the model. The “already predicted features” are those uncovered after training, together with their SHAP values, which may be positive or negative in relation to the outcome. PanomiX uses these SHAP values to set constraints, illustrating each feature's relationship with the predicted outcome. In ‘random constraints’ setting, the direction (positive or negative) of feature-outcome relationships is not predefined. In this case, users provide a table with two columns—“feature” and “final_association”—where “final_association” contains only 0 (meaning the relationship is unknown). PanomiX then applies random constraints using this information together with the “predicted outcome.” In the ‘constrained’ setting, monotonic constraints require a table with the same columns, but users assign 1 for a known positive relationship, −1 for a known negative relationship, or 0 if it is unknown. PanomiX merges this information with the “predicted outcome” from the trained model, ensuring predictions remain consistent with domain knowledge.

### 3.6 Comparing panomiX with other tools of multi-omics data integration

**Fig. 6:**
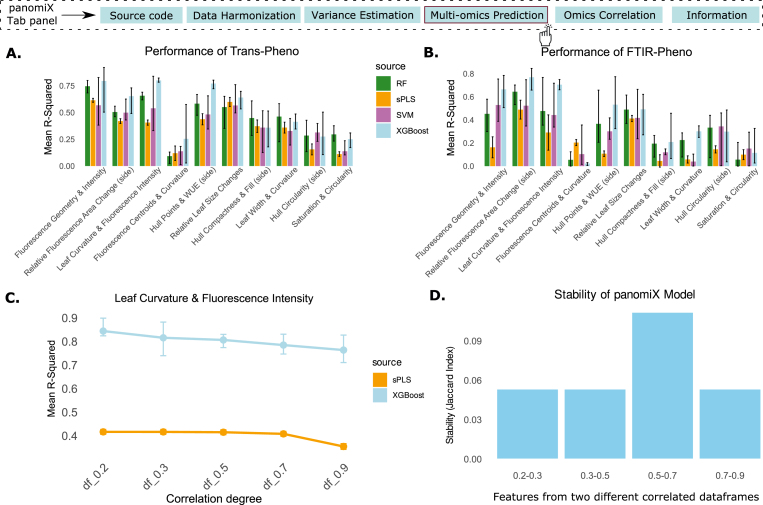
Multi-omics integration in panomiX and other methods. (A) Performance for transcripts-phenotype prediction. (B) Performance for FTIR-phenotype prediction. (C) Effect of multicollinearity on model performance (linear vs. non-linear method). (D) Robustness of feature selection for different thresholds of the feature collinearity in the panomiX XGBoost implementation.

To evaluate the efficiency, explainability, and stability of the panomiX multi-omics cross-prediction functionality, we tested it against mixOmics [32], a comparable state-of-art tool offering similar functionalities. Specifically, we compared the statistical methods offered by both tools as a main technique of data integration, namely the sparse Projection to Latent Structures (sPLS) of mixOmics with the XGBoost implemented in panomiX. We benchmarked both methods against standard implementations of Random Forest (RF) and Support Vector Machine (SVM) [49]. For that purpose, we conducted two cross-prediction analyses: a) transcriptomics data was used to predict phenotypic traits; b) the same phenotypic traits were predicted from the FTIR data. For each phenotype prediction, models were trained using four independent train-test splits (Fig. 6A and B).

To further investigate robustness, we compared panomiX and mixOmics (sPLS) on transcriptomics subsets with varying correlation coefficients (0.2–0.9), using one phenotype cluster (Leaf Curvature & Fluorescence Intensity) as a test case. Despite sPLS's known strength with highly collinear data, panomiX consistently outperformed it (Fig. 6C). We also evaluated feature importance stability across the correlation gradients by comparing the top 10 ranked features (Jaccard index). PanomiX consistently identified common features (Fig. 6D), indicating it effectively captures both linear and non-linear relationships in transcript–phenotype data while maintaining high predictive performance.

It is important to note that several tools exist for the integration of multi-omic data, each with different primary focus: *e.g.* DIABLO (which employs sPLS-DA from the mixOmics framework) [79] and MOGONET are primarily designed for classification [80], while MOFA can be applied to both classification and regression tasks [27]. While panomiX primarily excels in regression problems, it might be also deployed for classification. Thus to compare these methods, we performed a simple benchmarking for a two class classification task (control and heat treatment), using the default model parameters for both integration and prediction. For the analysis, transcriptomics and FTIR data were jointly used to predict treatment classes. Samples were grouped according to biological time points: no-stress (days T0), early heat treatment (days T1-T3), and recovery phase (days T8-T10). In each iteration, one time point was held out for testing, while the remaining time points were used for training. This leave-one-out procedure was repeated across all grouped time points to assess temporal generalizability. Statistical testing revealed no significant differences between panomiX and the other methods. Thus, the results demonstrate that panomiX performs comparably to existing approaches (Fig. S4).

### 3.7 Evaluating performance of panomiX across species and traits

To further validate and benchmark the performance of panomiX in other experimental setups other than time series and treatment contrasts, we utilized two publicly available multi-omic datasets for genetic diversity panels. The first dataset was obtained from Gemmer et al. [81] comprised eight agronomically relevant traits obtained from multi-year field trials and 128 metabolites profiled from the HEB-25 barley (*Hordeum vulgare*) nested association mapping (NAM) population, with 1307 individual lines. Using this metabolic dataset, we applied panomiX to predict each trait, without accounting for population structure during model training. panomiX outperformed the BayesB method for predicting the time to shooting (SHO) trait and showed comparable performance to BayesB for time to heading (HEA), grain yield (YLD), grain number per ear (GNE), and plant height (HEI). In contrast, BayesB had better performance for time to maturity (MAT), ears per m^2^ (EAR), and thousand grain weight (TGW) (Fig. S5A). These differences suggest that while panomiX effectively captures polygenic signals, accurate prediction for some traits in stratified populations (subgroups of related individuals with shared ancestry) may require explicit modeling of population structure, as done in the BayesB method. Performance differences may also arise from the underlying genome–trait relationship. For example, Bayesian regression is expected to perform well when the association between predictors and a trait is primarily linear or additive, while XGBoost will capture non-linearities, thresholds and interactions.

The second dataset Knoch et al. [10] features a large comprehensive multi-omics panel of spring-type oilseed rape (*Brassica napus*) on a diverse population with 477 lines. This dataset integrates high-throughput phenotyping, mRNA-Seq transcriptomics, and GC-MS-based metabolomics. We selected six phenotypic traits for validation: the manually determined plant biomass (fresh weight) and four image-derived traits — digital biovolume, plant height, compactness, projected leaf area and a color-related trait — measured at representative time points. For all six traits, panomiX consistently outperformed the RF-based regression models reported in the original study (Fig. S5B), achieving an average improvement of 12 % in the predictive ability (R^2^). Notably, several of the top-ranked transcripts identified in the original study were again detected by panomiX among the features with the highest SHAP values (Table S5), further supporting their relevance across different modeling approaches. These include C07p48260.1_BnaDAR (PRL1), one of the prime candidate genes reported to be associated with early vegetative biomass and growth-related traits, but also A03p39940.1_BnaDAR, annotated as an ethylene-responsive element binding protein, C07p57790.1_BnaDAR, a homolog of the Arabidopsis xyloglucan endotransglucosylase/hydrolase 17, and the two homeologs A05p28550.1_BnaDAR and C05p43970.1_BnaDAR, both encoding TRAF-like family proteins. Furthermore, several additional promising candidate genes were reported such as A10p28380.1_BnaDAR, showing homology to the Arabidopsis Succinyl-CoA ligase alpha subunit, a key catalytic enzyme of the citric acid cycle, as well as further putative xyloglucan endotransglucosylases/hydrolases such as A01p07860.1_BnaDAR and C01p08760.1_BnaDAR with putative function in the cutting and rejoining xyloglucan chains, which remodels the cell wall and allows cell expansion and growth.

## 4 Discussion

In this study we introduced panomiX, a user-friendly toolbox for multi-omics data integration. We demonstrated the versatility and robustness of panomiX on a unique multi-omic data set designed to identify transcriptomic and metabolic features associated with phenotypic traits. By addressing key challenges in normalization, variance analysis, and predictive modeling, we showed how panomiX enables extracting biologically meaningful insights from such complex datasets.

Our tool provided a robust workflow for transcriptomics and FTIR data, including tailored normalization and scaling of individual datasets. As shown in Fig. 3, stepwise transformations - here, from raw FTIR spectra to normalized outputs - improve data quality and interpretability through baseline correction and smoothing. This mitigates biases in raw datasets and ensures compatibility with downstream analyses such as PCA. PanomiX's PCA and ANOVA functions further standardize and visualize variance across separate and combined datasets, revealing consistent clustering patterns driven by time points and treatment conditions. These insights highlight the complementary nature of multiple omics layers, setting the stage for more targeted multi-omics analyses.

PanomiX implementation of XGBoost consistently outperformed sPLS, RF and SVM for the integration of our tomato heat stress datasets, showing higher accuracy, robustness, and feature stability. Its XGBoost-based framework effectively handles varying collinearity and identifies non-linear relationships, a key limitation in linear models. High Jaccard indices confirm feature stability across correlation gradients, while GO enrichment validates the biological relevance of identified predictors. Notably, the differential performance between control and treatment models underscores condition-specific mechanisms, exemplified by transcripts such as cytochrome *b*559 subunit alpha and phylloplanin in our experiment. These results highlight panomiX's capacity to reveal critical molecular interactions and support hypothesis-driven research.

By integrating transcriptomics, FTIR, and phenomics data, panomiX uncovers shared predictors - such as cytochrome *b*559 subunit alpha and specific FTIR spectra - indicating convergent biological mechanisms. These cross-layer insights reinforce panomiX's ability to reveal broadly relevant molecular features. Moreover, the option to add user-defined elements (e.g., heat-stress genes) illustrates its versatility for hypothesis-driven analyses. Identified interactions among kinases and stress-response transcripts emphasize the tool's value for mapping complex biological networks, and point to the need for evaluating both positive and negative feature associations when predicting phenotypic outcomes.

Compared to other multi-omics integration frameworks, panomiX is distinct in its focus on regression-based prediction of continuous phenotypes, whereas many established tools such as DIABLO or MOGONET are primarily for classification tasks. This feature expands its applicability to quantitative traits such as growth rates or stress indices, which are often more biologically informative than binary outcomes. It should be emphasized, however, that while individual models trained with panomiX are not directly transferable across experiments, the framework can be readily retrained on new datasets. Additional benchmarking on publicly available barley and oilseed rape population datasets further demonstrates this adaptability and supports the broader applicability of panomiX across different species, omics layers, and trait types. Beyond the prediction of agronomic/phenotypic traits, the use of SHAP values allows for the interpretation of model predictions by quantifying the contribution of each feature, thereby allowing to identify key genetic drivers linked to biological processes. This approach was exemplified in the oilseed rape dataset through the identification of promising candidate genes, such as PLEIOTROPIC REGULATORY LOCUS1 (PRL1), which has recently associated with actin microfilament integrity and cell morphogenesis [82]. Furthermore, additional transcripts with high SHAP values (Table S5) suggest biological relevance to the trait under study. These include several xyloglucan endotransglucosylases/hydrolases, which are cell wall remodeling enzymes involved in xyloglucan metabolism, and have found to be correlated with growth-related traits, including the projected leaf area, compactness, and early plant height [10].

As other statistical integration methods, generalization of the panomiX models is limited to datasets with the same variables measured. However, the applicability of the panomiX pipeline has been successfully demonstrated for various sources of variance; here complex time-series/treatment experiment, as well as a large-scale genetic diversity panel and a breeding population. While panomiX does not currently adjust for population structure or relatedness, its predictions are comparable with *e.g.* BayesB regression that includes this information explicitly in the model. In future implementations, incorporating a kinship matrix or population structure could further improve predictive performance in stratified populations. Additionally, environmental information could potentially be included as a monotonic constraint to guide predictions.

While panomiX offers significant advancements, several future challenges merit consideration. For example, in extending analyses to proteomics and metabolomics, peptide/protein ambiguities and metabolite identification uncertainties may affect the interpretation of features. Potential platform-specific biases and incomplete or uncertain annotations could influence pathway-level insights. Addressing these challenges through improved data curation steps, incorporation of databases, robust handling of missing values, and careful interpretation of predictive features will be important for broadening panomiX's applicability across diverse omics datasets.

## Code and data availability

The code for panomiX is freely available at https://github.com/NAMlab/panomiX-tool under the terms of the MIT license (also archived at Zenodo at time of publication: https://doi.org/10.5281/zenodo.15193421). The sequence data for this study have been deposited in the European Nucleotide Archive (ENA) at EMBL-EBI under accession number PRJEB85881 (https://www.ebi.ac.uk/ena/browser/view/PRJEB85881). Phenotyping and FTIR data as well as pre-processed inputs for reproducing the results of this article with panomiX are available at https://doi.org/10.5447/ipk/2025/3.

## Author contributions

A.S., J.S., D.P., K.P., D.K., and N.T. wrote the manuscript with input from all the authors. D.P. planned, conducted, and coordinated the experimental work for transcriptomics analysis, FTIR analysis, and phenotyping under J.S. and K.N. supervision. A.H. performed RNA sequencing, H.R., and L.B. performed FTIR analysis. A.S. implemented SHAP-based monotonic constraints for machine learning models. A.S. developed the toolbox under J.S.‘s supervision. A.S., and D.P. performed the data analysis under J.S.‘s supervision. A.S., and D.K. performed the comparative analysis of oilseed rape data under J.S.‘s supervision.

## Funding

J.S. and N.T. are funded by the Deutsche Forschungsgemeinschaft (DFG, German Research Foundation) under Germany’s Excellence Strategy—(EXC-2048/1–project ID 390686111). D.P. was funded by the Collaborative Excellence Grant of the Leibniz Association (No. K287/2019).

## Competing interests

The authors declare that they have no competing interests.
