# Supplementary material for: panomiX: Investigating mechanisms of trait emergence through multi-omics data integration
Source: Plant Phenomics. 2025 Oct 11;7(4):100131. doi: 10.1016/j.plaphe.2025.100131 (PMC13109294; doi:10.1016/j.plaphe.2025.100131)
Supplement: Multimedia component 6 [file mmc6.docx]

# **Supplementary Materials**

Table S1. The correlated matrix of all the 138 phenotypes we obtained from the IPK Phenosphere platform reduced to 27 uncorrelated phenotype clusters.

Table S2. Transcripts and FTIR features were consistently identified as top predictors across all three omics-specific models.

Table S3. Transcripts showed positive interactions with both the predicted features and the target phenotypes (scenario a).

Table S4. Transcripts showed interactions with both the predicted features and the target phenotypes from no constraints, random constraints, and monotonic constraints model (scenario b).

Table S5. Transcripts that were identified as top predictors across different traits in spring-type oilseed rape are listed, along with their corresponding SHAP values, which quantify each transcript’s contribution to the predictive models.


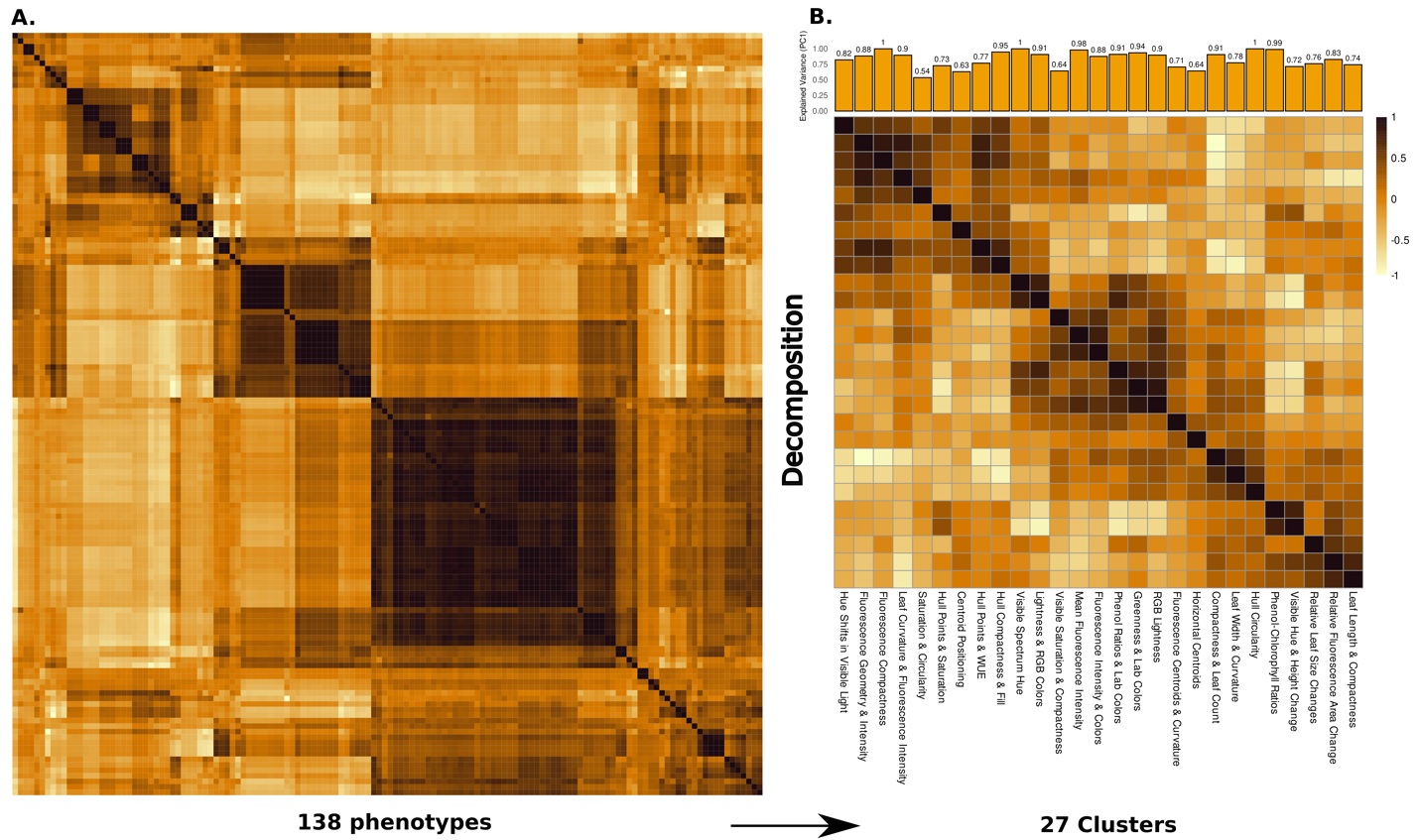


Fig. S1. (A) Correlated matrix of all the 138 phenotypes we obtained from the IPK Phenosphere platform. (B) Here we have obtained the cluster of correlated phenotypes using hierarchical clustering and then we used PCA based decomposition on each cluster. Total explained variance (for PC1) for each dataset has been illustrated in the top of the figure (B). On the bottom we represented the correlation plot of each decomposed phenotype cluster. More info (Table S1)


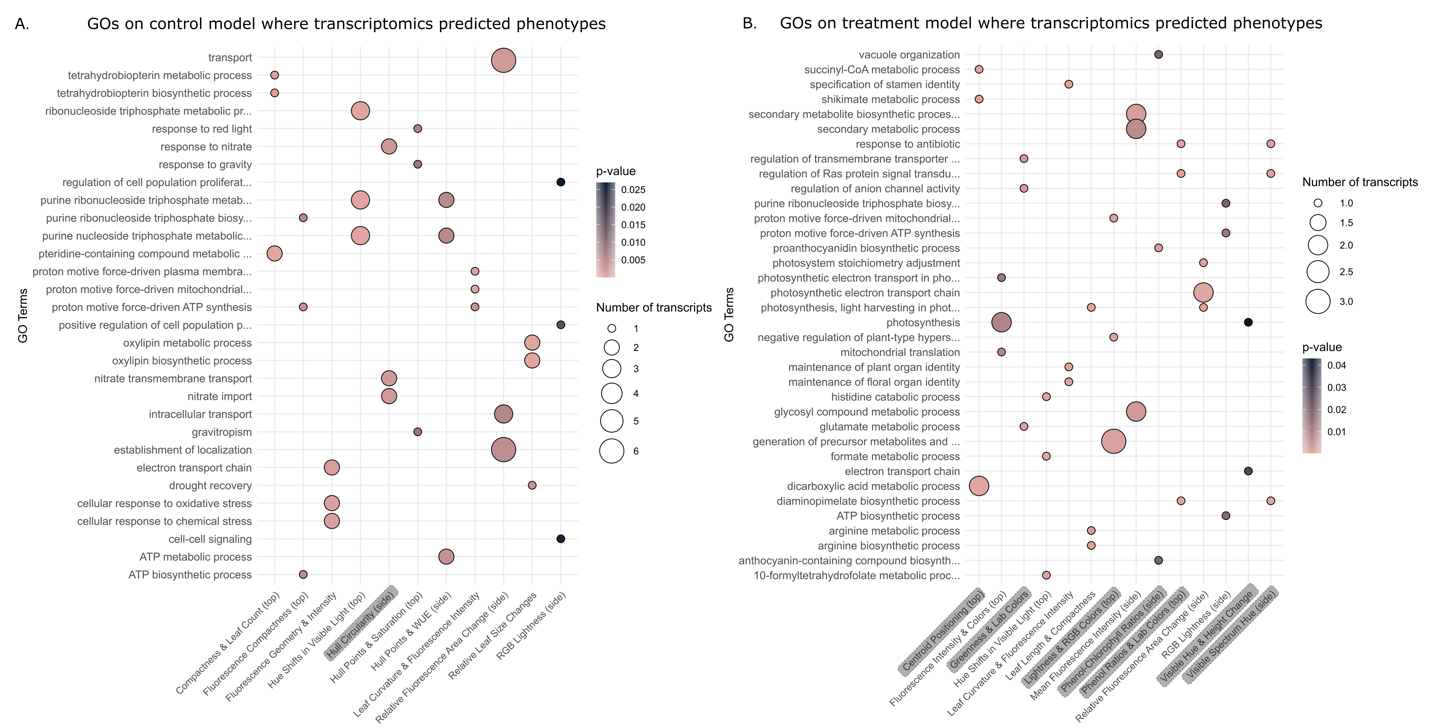


Fig. S2. (A) Top 3 gene ontology (biological processes) for the features selected from the control model (transcriptomics-predicting phenotypes) (B) Top 3 gene ontology (biological processes) for the features selected from the treatment model (transcriptomics-predicting phenotypes). Models shown with grey shading have a predictive performance of R² > 0.3 and replicate-model R² > 0.5. Models without shading have R² > 0.5 and replicate-model R² > 0.7.


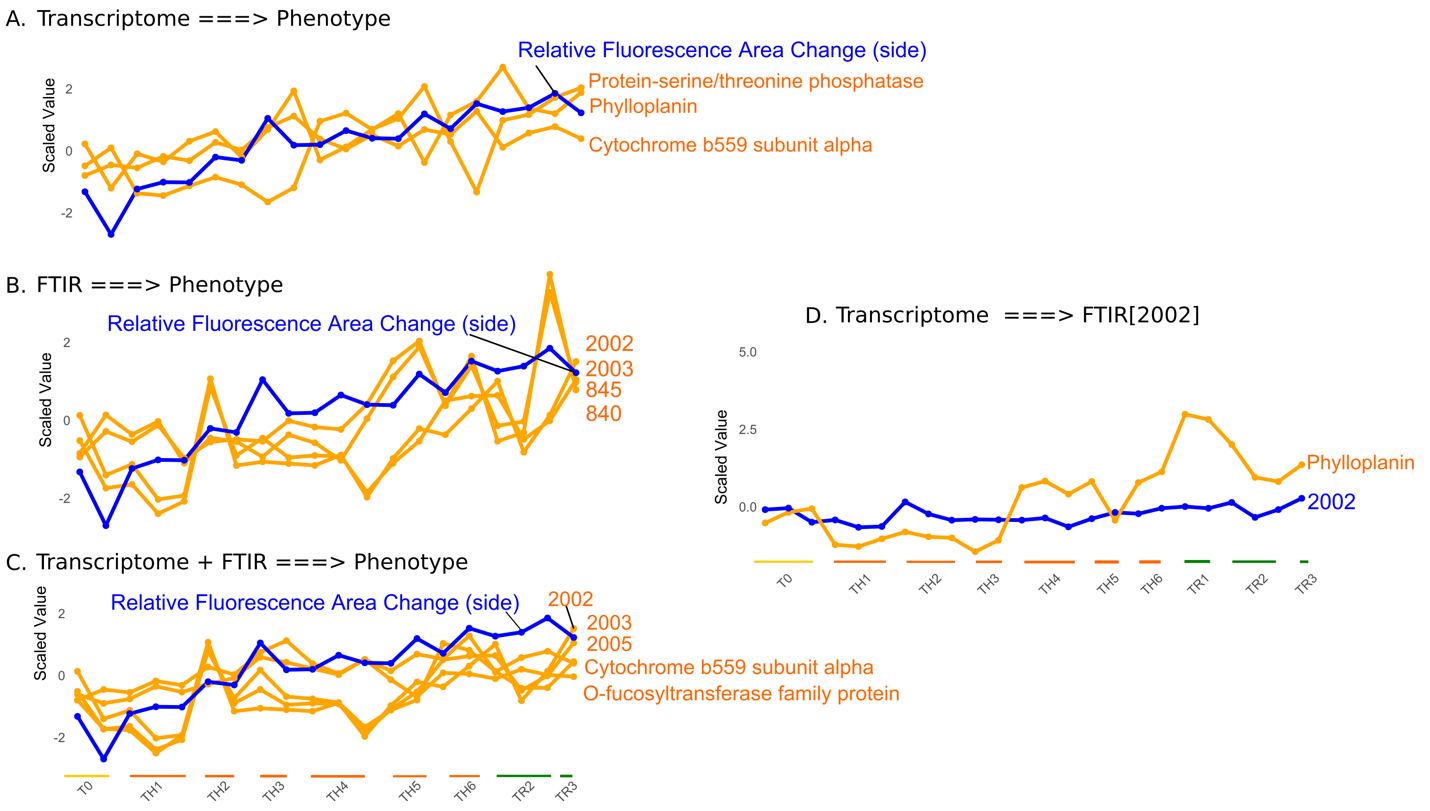


Fig. S3. Features selected from different integrative models, where important features are highlighted in orange and the predicted variable is shown in blue. (A) Expression profile for the Transcriptome predicting Relative Fluorescence Area Change (side) (B) Feature important for the FTIR predicting Relative Fluorescence Area Change (side) model (C) Feature important for the Transcriptome + FTIR predicting Relative Fluorescence Area Change (side) model (D) Expression profile for the Transcriptome predicting FTIR wavelength [2002].


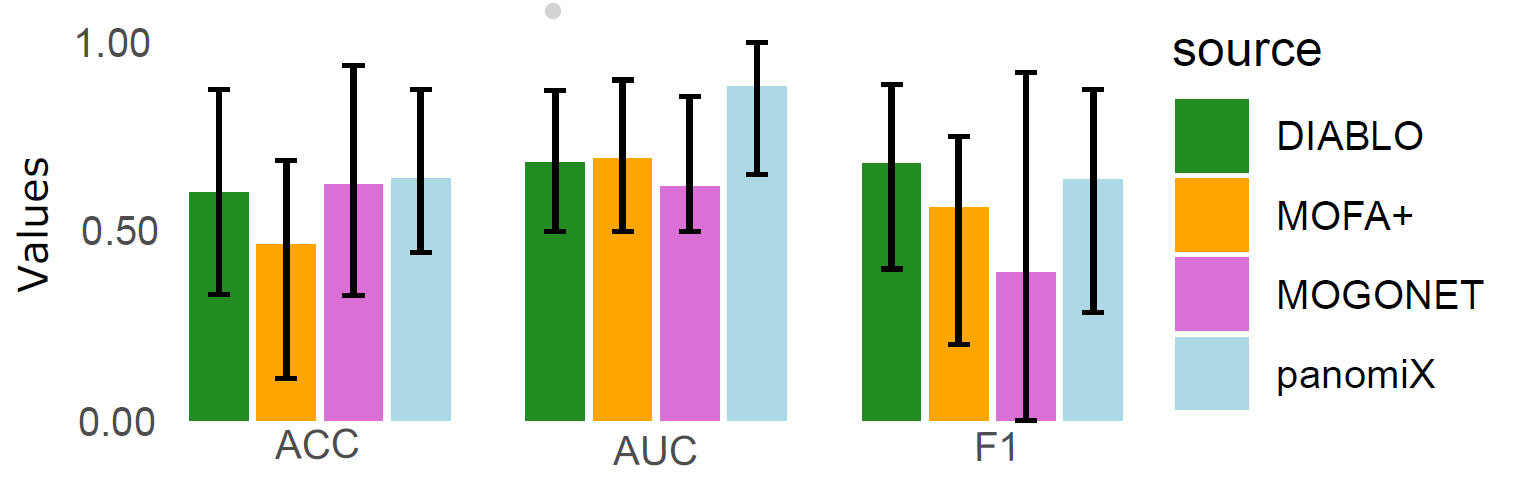


Fig. S4. Benchmarking panomiX on a classification task across time points. Three publicly available multi-omic integration tools- DIABLO, MOGONET, and MOFA+ were compared with panomiX. Test samples were grouped into three biological time points: no-stress (T0), early heat treatment (T1-T3), and recovery (T8-T10), with the remaining time points used for training. Each group was held out in turn for testing, and mean performance metrics, including AUC, accuracy (ACC), and F1 score, were calculated across all three time points.


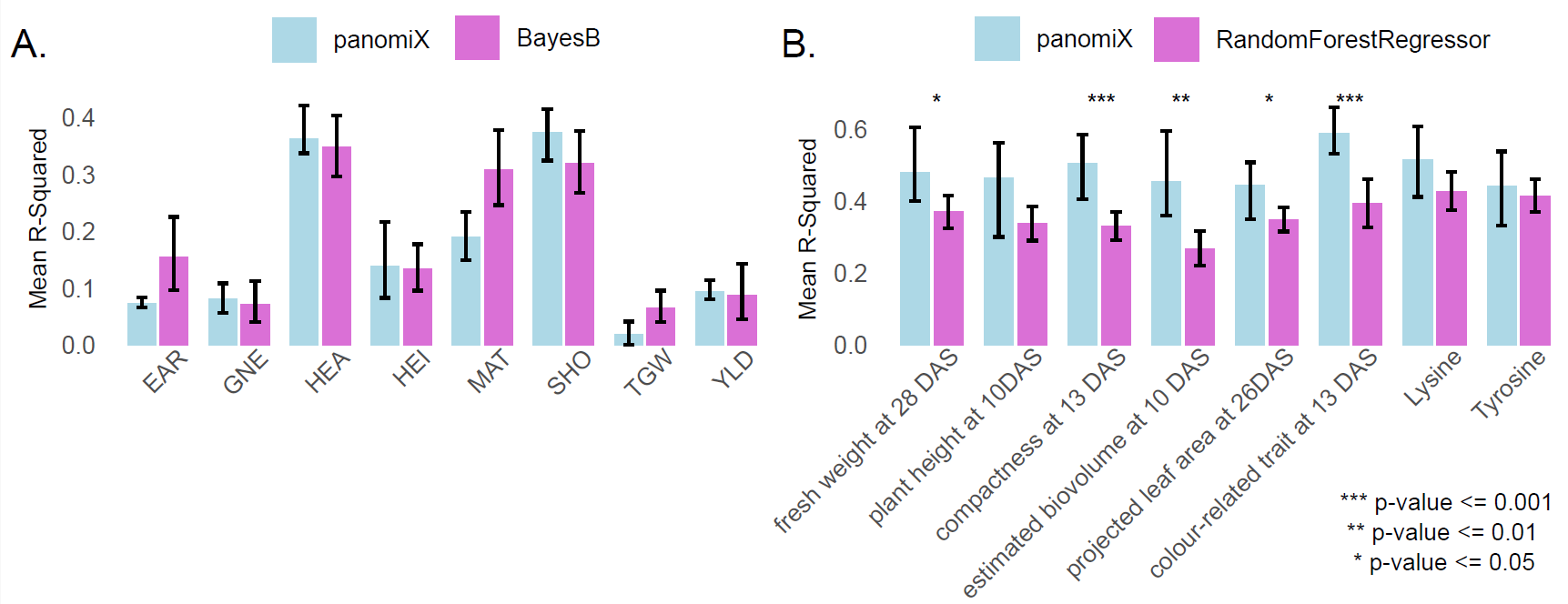


Fig. S5. Benchmarking the performance of panomiX across two multi-omics datasets. (A) Prediction of eight agronomically relevant traits in the HEB-25 barley NAM population using metabolites from 1,307 lines. Traits included time to shooting (SHO), time to heading (HEA), grain yield (YLD), grain number per ear (GNE), plant height (HEI), time to maturity (MAT), ears per m² (EAR), and thousand grain weight (TGW). PanomiX outperformed BayesB for predicting SHO, showed comparable performance for HEA, YLD, GNE, and HEI, and underperformed relative to BayesB for MAT, EAR, and TGW. (B) Prediction of six phenotypic traits in spring-type oilseed rape using transcriptomics data. In addition, the relative abundance of two metabolites, tyrosine and lysine, were predicted using transcriptomics data. PanomiX consistently outperformed Random Forest, with statistically significant (Welch’s t-test) improvements for fresh weight at 28 DAS (*P < 0.05), projected leaf area at 26 DAS (*P < 0.05), estimated biovolume at 10 DAS (**P < 0.01), compactness at 13 DAS (***P < 0.001), and color-related trait at 13 DAS (***P < 0.001).
